# Supplementary material for: Neuroplasticity Elicited by Modified Pharyngeal Electrical Stimulation: A Pilot Study
Source: Brain Sci. 2023 Jan 10;13(1):119. doi: 10.3390/brainsci13010119 (PMC9856550; doi:10.3390/brainsci13010119)
Supplement: Supplementary file 1 [file brainsci-13-00119-s001.zip › brainsci-2062155-supplementary.pdf]

Supplementary Figure 1. Pipeline of data pre-processing by Homer2.

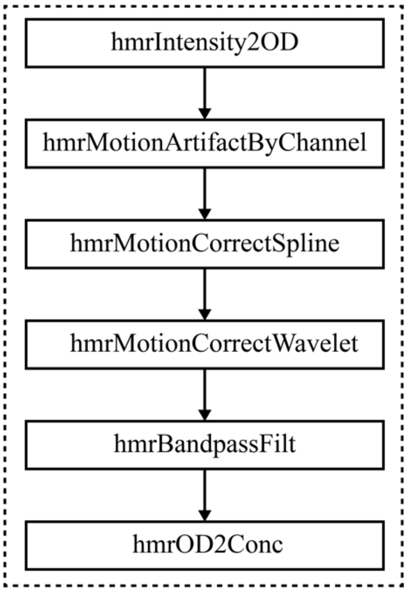

Supplementary Table 1. Channels excluded for Experiment 1.

Participant#	Excluded channel# (Experiment 1)
1	14
3	4，12
4	12
5	4，24，48
8	32
9	11，12，57
10	3，4，5，8，9，11，12，18，19，24，26，28，40，55
12	28
13	27，32，36，38，54，55，57
15	11，12，26，44，54，56，57
18	40
19	50

Note: Channel# 42, 43, 58, 59, 60, 61, 62, 63 located in occipital areas were excluded for all analysis, which is not listed in the table (the same in Supplementary Table 2).

Supplementary Table 2. Channels excluded for Experiment 2.

Participant#	Excluded channel# (Experiment 2)	
	Pre	Post
1	5，6，7，22，24	
3	12	
4	13，56，57	
5		12，13，14，15
6	23，31，33，34，36，50，51，56，57	12，31，33，35，36，40，41，50，51，54

7		12
